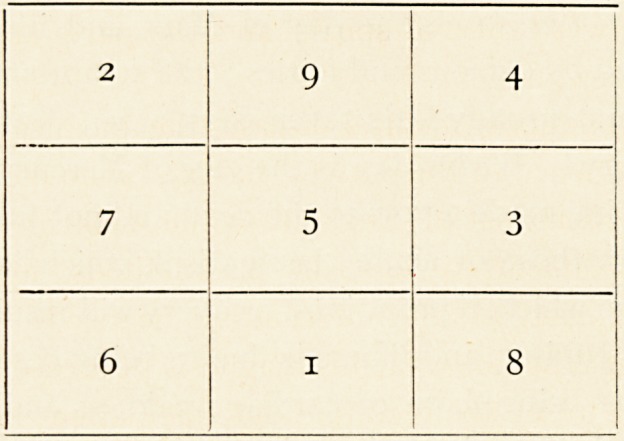# Oriental Medicine—Stray Jottings

**Published:** 1910-03

**Authors:** V. B. Green-Armytage


### ORIENTAL MEDICINE—STRAY JOTTINGS.

BY

V. B. GREEN-ARMYTAGE, I.M.S.

THE customs and people of the East are slow to move, but perhaps most of all is this observable in the practice of Oriental medicine. I have therefore attempted to put on paper a few examples of Oriental lore, with a view to interesting some of your subscribers. But should any reader be too critical, I would ask him to remember that in the East the system of Purdah, together with the machinations of superstition, make original investigation very difficult, however good one's linguistic powers may be.

In Northern India the native has a curious method of prophesying the sex of the unborn child: for at the seventh month many relations gather at the house of the future mother, and then a few drops of milk are squeezed out of her breasts on to a piece of yellow cloth; if, when it dries, it is white, a girl will be born; if yellow it will be a boy.

Child-birth itself is a great occasion, fraught with many superstitions; so perhaps an account of such an incident will not be out of place.

Immediately after the birth of the child the mother is given a small piece of metal copper to swallow, called "dumree," which is supposed to expel the placenta. After this she is given a dose of assafœtida, to prevent her catching cold and to relieve the after-pains; this drug being also affirmed to be a stimulant. The midwife, like the gipsy of the West, is wise in her generation, for she demands at this stage something shining, such as a silver or gold coin, just to touch the umbilical cord with, "for luck;" and then she proceeds to deal with it in the usual way—appropriating the coin for herself. In the East—unlike the West—the placenta is not a thing despised, but is buried with due state, along with a sacred leaf and small coin, to propitiate the gods; and the knife that cut the cord is kept sacredly for forty days, alongside the mother and child wherever they go or move, for it has a certain power against all ghosts and evil spirits.

During the puerperium the mother is looked after with great care, and for forty days she is only allowed to drink boiled water, in which a red-hot horse-shoe has been allowed to cool. The wisdom of this course will be easily seen. However, she and her child have ever to be on guard against evil influences. No dog or cat must come near her, and more especially the latter, as it is regarded as a witch. Moreover, any stranger coming to the house has to throw some myrrh on the fire to ward off all evil spirits from the mother and child. As soon as the child has been washed it is brought and presented to its relations, when, if it belong to a Mussulman family, the "Azan," or call to prayer, is pronounced in his left ear, and the "Tukbeer," or creed, into his right. Baptism soon follows, and is usually rather a gamble, for one of the family will open the Koran at any page, and then the first letter of the first word on the page is chosen to construct the child's name on. Like the Jews, every Mussulman is circumcised. The occasion is usually one of much hilarity for the family, but of suffering for the patient, as the Koran law decrees that it must be done between the ages of seven and fourteen. The boy is placed on an earthen drum and is given bhang (Cannabis Indica) to drink. He is then engaged in conversation, whilst the homely barber deftly wields the razor. After the operation a peacock's feather and a copper ring are tied round his neck, to ward off evil spirits, and the parts are dressed with powdered benzoin.

For the commoner medical diseases the uneducated Indian has implicit confidence in the native "hakim," who corresponds very much to the herbalist of England or the medicine man of Zululand. It is difficult to describe his methods, for his eye and conversation elicit all the information he needs for treatment. Once I asked a hakim what his diagnosis of a case was, and how he was going to set about to find out, the patient being delirious. His answer was: "Sir, God knows his disease; we only know whether it is a hot or a cold disease, and according to which I find or think it, I shall give him of my drugs." This will give you an idea of their *modus operandi*. But I may say that often their medicine has excellent results, and they, moreover, know their limitations, for they send their surgical and unsuccessful cases to the civil surgeon, partly, no doubt, because they can obtain no more money.

One rather curious mode of treatment is to apply to a septic ulcer an oil expressed from the head of a cobra; and another, for impotence, a condition ever in the East calling for treatment, is to take the penis of a crocodile (these brutes abound in the rivers), dry it in the sun, and then powder it finely. The powder thus obtained is a very valuable and precious remedy, and fetches large sums of money for a dose of a few grains.

Another is to apply to every sore, or ulcer, or boil, a compress of sugar and soap—which readily cures. This, of course, is a remedy I have seen very commonly used in the West Indies, and is interesting in view of Sir A. E. Wright's work on capillary transudation.

But perhaps the most extraordinary and interesting thing of all is the faith in the power of charms to heal. I have spoken in a previous article, of a so-called snake bite cure, but this is as nothing, compared to an amulet which was sold to me as a certain cure for fearsome hemorrhoids or confluent small-pox. It consisted of a metal disc, on which were scratched the following figures and lines :—

| | | |
|---|---|---|
| 2 | 9 | 4 |
| 7 | 5 | 3 |
| 6 | 1 | 8 |

the figures of which, added up vertically, diagonally or horizontally, make the same total. I was assured that all that was required was to wash the disc two or three times a day in water, then drink the water, and mutter, "La-il-la-hah, il-lul-ha-ho, Mohamed, oor Rasul-Ullahah" ("There is no other God except the one true God, and Mohamed is the prophet of God"), and that the utterer would soon be well! There are a great variety of these discs—all varying in potency, and covering the whole gamut of diseases.

Nor should it be thought that prognosis will offer any difficulty to these astute fellows, for they have a very definite system of application, which I will explain. First, they inquire on what day the man went sick. If he cannot say, they take the number of letters in his, or his mother's, name and divide it by seven ; if one remain he must have been taken ill on a Saturday; if two, on a Sunday, and so on. Then the procedure is as follows. If on *Saturday*, as it is Saturn's day, the disease must be due to heat of the blood, or a malignant eye, and must last seven days; and the only treatment is for his friends to give propitiary alms to fakirs, and to wear certain amulets and drink the water thereof. If on *Sunday*, as it is the day of the sun god, it is due to the evil eye of some green-complexioned woman with whom he has taken food; the symptoms are those of anæmia and extreme debility, and are treated by iron water and "cold" sponges. If on *Monday*, the day of the moon god, it is caused by catching cold after bathing or exertion; the symptoms are those of acute hepatitis, and are treated by "hot" medicine and local applications. *Tuesday* is the day of Mars, and illness is due to being attacked by demons and fairies; the symptoms are those of peritonism, and usually lasts seven days, when death or life will win the victory! *Wednesday* is the day of Mercury, and disease is due to having made a vow to the dead and not fulfilled it; the symptoms are those of acute rheumatism, which lasts but nine days, and from which, if propitious, recovery will result. *Thursday* is the day of Jupiter, and illness is due to being beset by a fairy; the symptoms being those of cardiac weakness and indigestion; for which little food is to be taken. You will see, therefore, that Eddyism and Easternism are not so very far apart!

Amongst the lower classes of the people of India, surgery, except in the use of the bluntest knife, is almost unknown; and, indeed, it is their horrible, conservative fatalism that is one's greatest bugbear. Many times I have seen compound fractures only admitted to hospital either *in extremis* or with peripheral gangrene, the relatives having done nothing, perhaps for a week or more, but apply neem oil.

So far I have spoken only of India; but before finishing, I should like to mention two customs I have recently observed in Upper Burma—the one horrible, the other almost rational. The first is the Burman method of helping a difficult or prolonged labour, by merely jumping on the abdomen of the woman as she lies on the floor; or massaging the abdomen with his feet, the results being often terrible to both child and mother. The second is their specific for cholera, which they suppose to be due to the evil influence of a spirit; a bell is rung, and then all the villagers collect and rush to the tops of their reed houses and make a tremendous commotion, beating gongs and striking the roofs with a sort of native broom. This, they affirm, drives the evil spirit from the house and village, and certainly on occasion it has some effect; probably owing to the fact that they are all so elevated and engrossed in pursuit of the "Nat," or spirit, that little opportunity is afforded for the despondent and crushing effects of the cholera.

The story of plague is another instance of fatalism or superstition almost incomprehensible in the West. Quite recently, I remember, over a quarter of a million Mussulmans died, in one province alone, because they refused to leave their infected villages, on the ground that the Koran forbids a Mohammedan to flee from the wrath of God.

The natives swarm and herd together in enormous numbers amid unclean surroundings, and the B. Pestis takes its corresponding toll; but "kismet" is the only answer. Discouraging indeed is the work of the plague officer in districts as big as the County of Gloucester. Oft-times he is met with the tale, concocted by agitators, that plague is no disease, but a devilish contrivance of the Government to reduce the population, and this slander is believed. Indeed, the natives of India and Burmah view with the greatest abhorrence and suspicion such things as precautionary measures, segregation and disinfectants. Verily, *sunt lachrymæ rerum et mentem mortalia tangunt.*

## Figures and Tables

**Figure f1:**